# Patellar tendinopathy: an overview of prevalence, risk factors, screening, diagnosis, treatment and prevention

**DOI:** 10.1007/s00402-023-04998-5

**Published:** 2023-08-04

**Authors:** Andreas Theodorou, Georgios Komnos, Michael Hantes

**Affiliations:** Faculty of Medicine, University of Thessaly, 41500 Larissa, Greece

**Keywords:** Patellar tendon, Jumper’s knee, Patellar tendinopathy

## Abstract

Patellar tendinopathy (PT), or jumper’s knee, is an overuse injury that occurs in professional, as well as recreational, athletes. This condition is a noncontact injury, typically characterized by gradually increasing pain in the patellar tendon. It is prevalent in participants of several sports, but it occurs mostly in jumping sports. The diagnosis of PT is primarily clinical; however, imaging techniques can be useful as well. Risk factors differ between sexes, playing conditions, the kind of sport, playing level and personal characteristics. Screening is an essential tool to assess PT. This condition affects athletic performance and often persists for years. The use of preventative methods is imperative because of the persistence of this condition, especially in elite athletes who sometimes end their career after long and failed treatments. There are a wide variety of treatment and rehabilitation options available, the majority of which are non-operative, such as eccentric exercises, cryotherapy, platelet-rich plasma (PRP) injections, and anti-inflammatory strategies. If conservative treatment fails, surgery is the next most preferable step. Even though there are many surgical treatment methods, there is no clear evidence on what is the most effective approach to address PT. Taking this into consideration, as well as the extent of this clinical entity, novel therapeutic techniques, as well as screening and prevention methods, are expected to emerge in the near future.

## Introduction

Patellar tendinopathy (PT), despite its frequency and clinical importance, remains a real challenge for every sports physician, mostly because of its persistence [1, 2]. According to a prospective study involving elite athletes competing in football (soccer), long-distance running, volleyball, orienteering, basketball and ice-hockey, more than 50% of the athletes with PT were forced to retire from active sport [3]. High prevalence rates of PT are especially present in jumping athletes [4, 5].

Inflammation of the tendon could occur when there is overuse during athletic activities, although this can be return to normal when the load is adjusted. However, when high load persists, the injury can become chronic [6, 7].

For PT, several evidence-based nonsurgical and surgical treatment options and diagnostic pathways are presented in the literature [1, 8–10].

The aim of this literature review is to characterize typical symptoms, provide an overview of available risk factors, diagnostic techniques, screening, treatment and prevention options.

## Methods

The electronic database PubMed was searched in March 2021. Considering the developed promoted by scientific progress in imaging, non-operative treatment and surgical techniques, the search was conducted from January 2001 to March 2021. The following key words were used alone and in combination: “patellar tendinopathy”, “jumper’s knee”, “prevalence”, “risk factors”, “screening”, “diagnosis”, “treatment” and “prevention”.

All retrieved article (*n* = 747) abstracts were screened for this review, and all the irrelevant papers were filtered out. Furthermore, reference lists from the selected studies and review articles (*n* = 84) were assessed for eligibility, and articles published before 2001 (*n* = 14) were added. During the final processing of the manuscript, another 4 articles were identified as relevant to our review and were added.

## Results

### Prevalence and risk factors

There are many causes and possible risk factors described in the literature that could lead to PT [11]. There are also controversial reports on some of the described risk factors.

According to Lian et al. the prevalence rates of PT in professional volleyball and basketball players are 45% and 32%, respectively, whereas the overall prevalence in elite players from nine different sports is 14.2% [4]. Van der Worp et al. reported that the prevalence of PT in their study population, which included volleyball and basketball athletes, was 18.6%, with 12.3% of the subjects diagnosed with unilateral PT and 6.3% with bilateral PT [11]. Zwerver et al. studied a population of 891 male and female non-elite athletes who played in seven popular sports; they concluded that the overall prevalence of PT was 8.5% (Table 1). The highest prevalence was in volleyball players (14.4%), followed by handball (13.3%) and basketball (11.8%) players, track and field athletes (6.9%), and then field hockey (5.1%), korfball (4.8%), and soccer (2.5%) players [5].

**Table 1:** Prevalence and risk factors of PT

Authors	Prevalence	Risk Factors
Lian et al. [4]	Volleyball 45% Basketball 32% Overall prevalence in these sports: 14.2%	Male gender High weight Tall height Increased number of weight and jump training Increased number of sport-specific training in basketball
Zwerver et al. [5]	The overall prevalence of PT is 8.5% Volleyball: 14.4% Handball: 13.3% Basketball: 11.8% Track and field: 6.9% Field hockey: 5.1% Korf-ball: 4.8% Soccer: 2.5%	Male gender Participating in sports Increased number of training hours Playing surface Young age Higher BMI Tall height
de Vries et al. [8]	Overall prevalence in study’s population (Volleyball and basketball): 13%	Male gender Heavier load on the patellar tendon BMI is not a risk factor Age is not a risk factor
Van der Worp et al. [11]	Overall prevalence in study’s population (Volleyball and basketball): 18.6%	Male gender Increased number of training hours Playing at the national level compared with playing at the regional level Playing volleyball compared with playing basketball Playing position in volleyball but not in basketball Higher age Playing surface BMI and waist-to-hip ratio were not associated with PT

Zwerver et al. identified that participation in sports is a risk factor for PT [5]. Furthermore, it could be assumed that the number of training hours is an additional risk factor for developing PT, because the elite athletes who trained for more than 12 h per week developed PT more frequently in comparison with non-elite athletes who practiced 4 to 5 h weekly, as has been reported by some studies [5, 12]. Similarly, Sprague et al. demonstrated that increased sports participation was a risk factor for PT [13].

Being more youthful and taller could be additional risk factors, according to Zwerver et al. [5]. However, these findings were not confirmed in some other studies [14–17].

In an online survey which included 2224 subjects, the identified risk factors found for PT included playing at the national level, male gender and playing volleyball (in comparison to basketball) [11]. This difference between volleyball and basketball players could be explained by the fact that professional volleyball players jump higher than professional basketball players, as measured in a drop jump task [17]. Specifically, volleyball players, such as outside hitters and middle blockers hitters, had an increased risk when compared with setters [11]. As for the basketball players, no risk factors related to playing style were found [11]. The personal monitoring of athletes according to individual risk factors may help prevent PT [8]. In addition, because the highest prevalence of PT in volleyball athletes concerns those who play as outside hitters or middle blockers/hitters, a change in playing position to libero/opposite hitters or setters could be an option [8]. In the same survey, the prevalence of men with PT (25.3%) was significantly higher in comparison to women (13.1%) [11]. The same findings were reported by two other studies, demonstrating that men’s odds when it came to PT are more than twice as high as in the opposite sex [5, 8]. This can be explained by the fact that women’s patellar tendons are exposed to lower forces because they have less quadricep strength and an inferior jumping capacity [5]. In addition, volleyball players had higher prevalence rates of PT than basketball players: 20.1% and 15.2%, respectively. Moreover, players playing with the national team had twice as high a prevalence of PT as compared with those at regional level [11]. This can be explained because playing at a higher level requires a heavier load being placed on the knee more frequently, because players at the elite level have stronger muscles and jump higher [11, 12]. A heavier load in jumping sports was also demonstrated as a risk factor for PT by other authors. [8, 13]. In the meta-analysis study by Sprague et al., jump height was also described as a risk factor [13]. Although Cook et al. identified that estrogen can exert protective functions on women’s tendons, other studies have indicated that estrogen can inhibit exercise-induced collagen synthesis, which leads to a lower rate of tendon tissue repair [16–19].

The playing surface is described also as a risk factor. Players who played on concrete had a prevalence of PT of around 38%, in contrast to the approximately 20% in athletes who played on other surfaces [11] (Table 1). Bahr and Reeser showed that the prevalence of PT is much lower in beach volleyball players [20]. This conclusion suggests that a softer playing surface, such as beach sand, reduces the risk of PT [11].

Additionally, in a study including 105 subjects, 52 with PT and 53 without, the lever arm ratio and moment arm ratio from lateral radiographs were significantly different between the two groups. According to findings from the radiographs, the patellar tendon exhibited smaller lever and moment arm movements in patients with PT when compared with patients without PT [21]. In their study, Dan M.J. et al. demonstrated that physicians can identify patients at risk through simple radiographic means, because these variations may lead to a greater force expended through the patellar tendon in patients with PT [21].

A higher BMI was also identified as a potential risk factor for PT in some studies, but this was not confirmed by others [5, 8, 22].

In their study, Bisseling et al. evaluated whether the landing strategy differed amongst healthy volleyball players and volleyball players with PT, to identify if the landing strategy is a risk factor in developing PT [23]. Interestingly, they concluded that asymptomatic players used a landing technique to avoid high patellar tendon loading, in contrast to symptomatic players who used a stiffer landing strategy, which the authors identified as a possible risk factor [23].

Finally, according to Visnes et al., the presence of hypoechoic areas and neovascularization among asymptomatic athletes represented a risk factor for developing PT as well [24].

### Diagnosis and screening

#### Clinical symptoms

Most of the athletes with PT present to physicians with well-located anterior knee pain [1, 25]. The pain is often increased by exercise, or occasionally, by prolonged knee flexion. Furthermore, in many cases, pain can start insidiously. Moreover, patients often associate the pain with a period of increased sport activity. In mild cases, pain is only present when performing sport activities, whereas when the condition progresses, they often complain about pain at the beginning or throughout the exercise. Additionally, in severe cases, patients report pain in performing daily activities, or even when at rest [1]. When patients with PT were instructed to execute a decline squatting exercise, they could only perform a limited number of decline squats without experiencing pain [26]. Although there are many scoring systems described in the literature, the VISA score seems to be the most widely accepted [25]. The VISA-P and VISA-A questionnaires include eight questions. Six questions are related to the pain experienced during a range of everyday activities, and the other two deal with the ability of the athlete to perform sports activities. The maximum VISA-P and VISA-A score for an asymptomatic athlete who regularly exercises is 100 points, and the theoretical minimum is 0 points [27].

#### Imaging

**Fig. 1:**
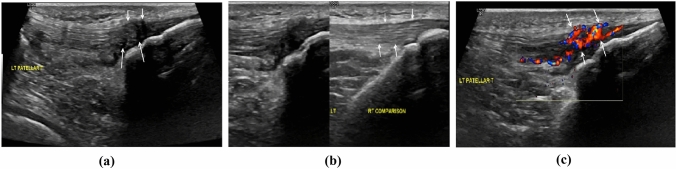
Ultrasonography images of the left patellar tendon, in a middle-aged elite athlete with persistent anterior knee pain. **a** Longitudinal B-mode image showing the inhomogeneous echogenicity in the distal anterior tendon in keeping with degeneration (short arrows) and microtears in the posterior aspect (long arrows). **b** On the right side of the image, the normal right patellar tendon is shown for comparison (arrows). **c** Longitudinal color Doppler image shows florid neovascularity in the degenerated foci (arrows). (Courtesy: Dr. G. Kakkos and Prof. A. Karantanas, Dpt. of Medical Imaging, Heraklion University Hospital)

**Fig. 2:**
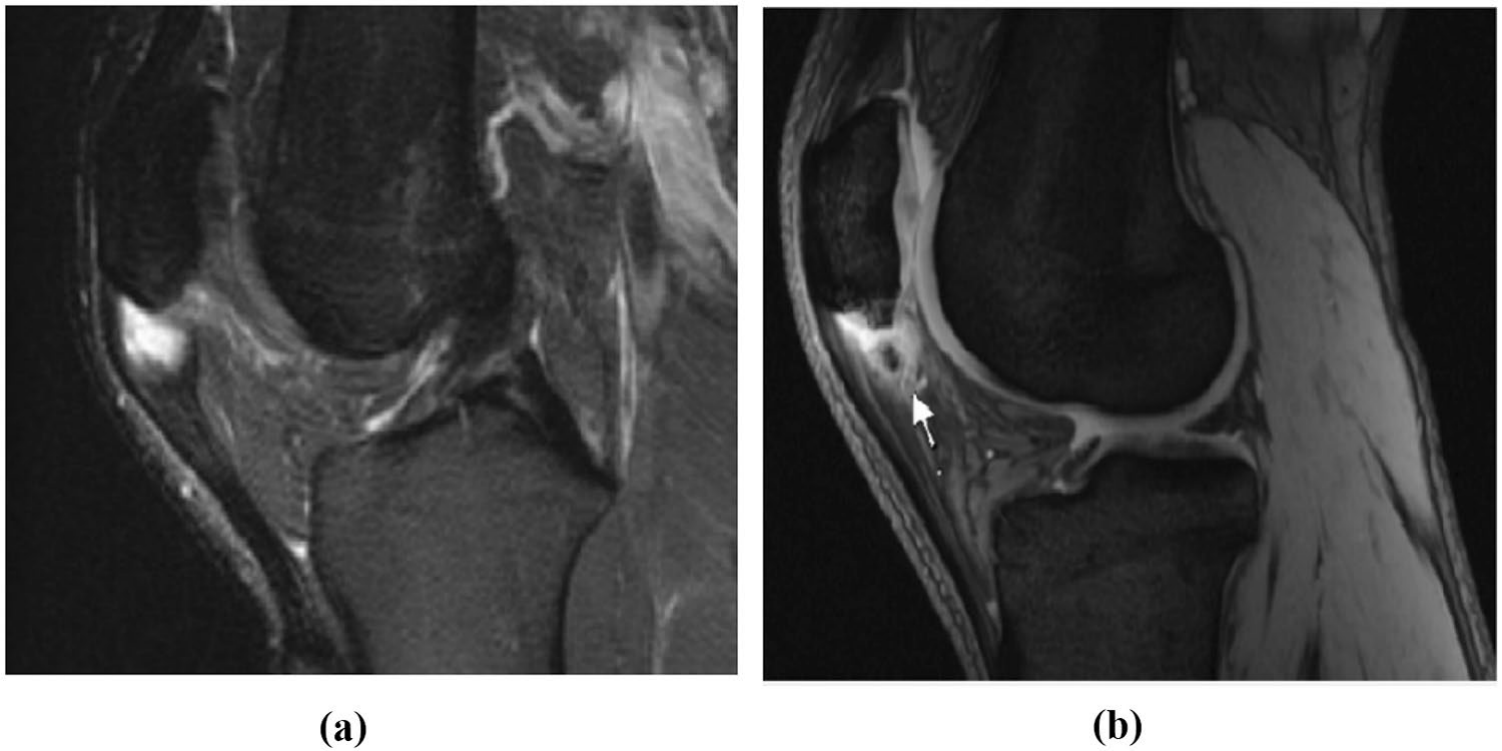
**a** T2-weighted MRI image of the left knee, in a young elite athlete with PT symptoms. It demonstrates an increased signal interposed in the patellar tendon. **b** T2-weighted MRI image of the left knee, in a young elite athlete with PT symptoms, showing a free bone formation in the mass of the patellar tendon

Ultrasonography (US), Doppler ultrasonography, MRI and X-rays are often used by physicians who deal with patients with PT. Plain X-rays can be used to detect correlated bone abnormalities, but MRI and US procedures are more useful due to the possibility of obtaining detailed visualization of the tendon [1]. Khan et al. described a good correlation between MRI and US for PT at histopathological findings on surgical biopsies [28]. US can detect a hypoechogenic zone often related with tendon thickening in PT patients [29]. Hypoechoic lesions are usually found in the posterior portion of the patellar tendon, near to the inferior pole of the patella [30]. Using Doppler ultrasonography, physicians can detect neovascularization and increased blood flow, which are usually present in symptomatic PT [31, 32] (Fig. 1). Additionally, MRI can detect PT as a thickened tendon with areas of increased signal intensity [29]. Furthermore, partial tears in PT patients are difficult to identify, but they are best demonstrated on T2-weighted images with high signal intensity [33] (Fig. 2). Both US and MRI have advantages and disadvantages. On one hand, US lacks the ability to rule out intra-articular abnormality and it depends on the experience of the operator. On the other hand, MRI can detect intra-articular pathology, but it is not always available, costs more and is time consuming [30]. Finally, the sensitivity and specificity of US for PT are 58% and 94%, in contrast to 78% and 86% for MRI, respectively [34].

#### Screening

Ultrasonography of asymptomatic tendons can be useful to predict which athletes will develop PT [35]. Fredberg and Bolvig examined 54 professional soccer players before and after a single season, using ultrasonography of the ankle and knee. Patients with asymptomatic hypoechoic regions in the patellar tendon at the start of the season had a significantly greater risk of developing symptomatic PT during the season, in contrast to those without hypoechoic regions: 17% of the former group developed PT, as compared to none in the other group [35]. Visnes et al. also insinuate that the presence of hypoechoic areas and neovascularization among 141 asymptomatic athletes at the beginning of their study represented a risk factor for developing PT. There were structural changes observed in the US examinations from the beginning in more than half of the tendons who later developed PT symptoms and 83% of the tendons at the time of diagnosis. Therefore, structural changes preceded symptoms in most of the patients. Nonetheless, two-thirds of patients who exhibited hypoechoic areas in their baseline US images did not develop symptoms during their 1.7-year observation period with intensive training. Thus, they concluded that they cannot recommend routine ultrasound screening programs to prevent PT [24].

Fazekas et al. claim that VISA-P scores may be useful as a screening tool for identifying the presence of hypoechoic areas in ultrasonography of the patellar tendon. In their study, which involved 31 asymptomatic athletes, the VISA-P scores were significantly lower in US exhibiting hypoechoic regions than in those without hypoechoic findings in the right knee, but not the left knee. Furthermore, Fazekas M.L. et al. compared the prevalence rate of hypoechoic areas between men and women in their study group; they found no significant differences in either the right or left knees [36].

Gisslén et al. examined 22 elite high school volleyball players (44 patellar tendons) by US and power Doppler (PD), and they continuously evaluated them clinically and with the use of US and PD during 3 school years [37]. This study has indicated that if clinical examination and US of the patellar tendon are normal at the start, there is a low risk of sustaining PT, despite exercising 13 to 15 h weekly for 3 school years. This is supported by the fact that in their study, among 27 tendons that were normal at the start, only 2 developed PT during the 3 years of intensive training and playing volleyball games. Furthermore, another finding of the same study was that if jumper’s knee was clinically diagnosed at the start, in most of the tendons, the symptoms remained [37].

### Treatment

Treatment of PT can be non-operative and operative. Conservative treatment is recommended as the first step, according to the literature.

#### Non-operative treatment

Conservative treatment for PT consists of many methods, but the most popular are eccentric exercises [25, 38]. Other non-operative treatment options include platelet-rich plasma (PRP) injections [9, 39–42], cryotherapy [43, 44], patellar strapping [45–51], NSAIDs [52–54], corticosteroids [35, 54–58], aprotinin injections [59, 60], sclerosing injections with a chemical irritant [61–66], glyceryl trinitrate (GTN) patches [67] and lastly, extracorporeal shockwave therapy (ESWT) [68].

#### Eccentric exercises

**Fig. 3:**
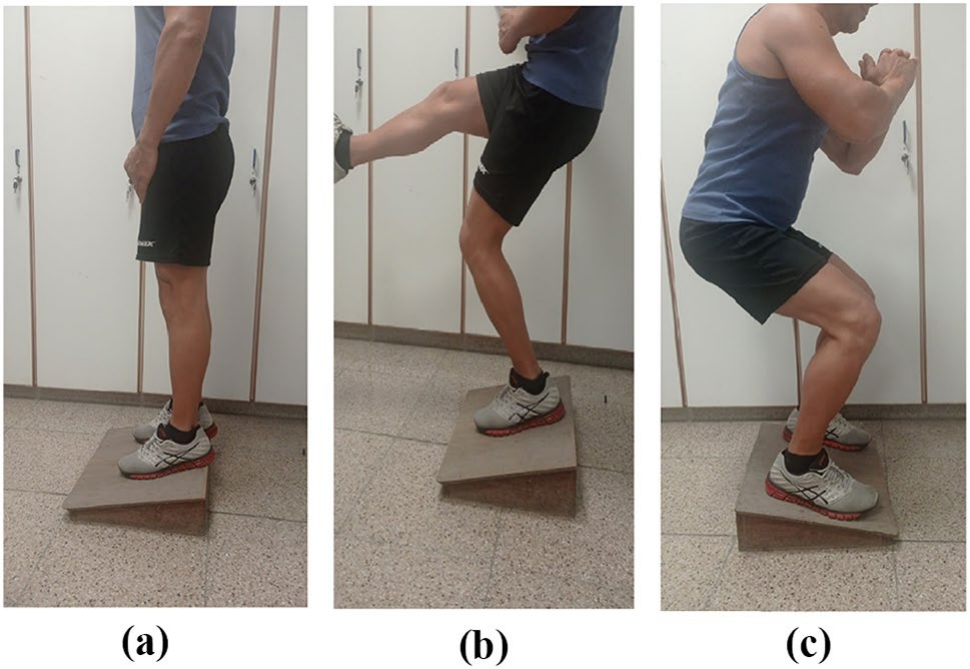
This figure shows an athlete performing an eccentric decline squat

In the literature, most studies suggest that eccentric training may have a positive effect on the treatment of PT. There are various eccentric exercise programs such as drop squats, squatting on a decline board, squatting on level ground, exercising until tendon pain, training until just before the onset of pain, exercise that involves loading in the eccentric phase only or both phases, and progressing with speed then loading or simply loading [38]. Young et al. suggest that an eccentric decline squat protocol offers better results at 12 months when compared to a step eccentric protocol for volleyball players with PT; the first group showed a 94% chance of a positive result, in contrast to 41% in the step group [69]. The decline squat was performed using a single leg on a 25° decline board (Fig. 3), with some level of discomfort [38, 69]. Johnson and Alfredson indicated that at 12 weeks, athletes with PT who were following a protocol with eccentric exercises improved significantly, in contrast to those who were following a concentric exercise protocol [70]. In the study by Visnes et al. [71], eccentric exercises were found to have no effect on knee function after a 12-week program when followed simultaneously by volleyball athletes who continued training and competing. In contrast, other studies have suggested that when athletes with PT continued to compete in sport, they benefited from an eccentric exercise protocol [57, 69, 72, 73]. When an eccentric exercise protocol was compared to surgical treatment (open patellar tenotomy), at 12 months, there was no significant difference between the two groups [74]. According to the same study, the treatment of PT should start with an eccentric exercise protocol for twelve weeks before taking into consideration an open patellar tenotomy [74].

According to van Ark et al., the current eccentric exercise protocols that are used in-season result in increases in pain [75]. In their study, which included a treatment program with isometric and isotonic exercises in 29 athletes with PT, both exercise programs were shown to reduce pain from PT for athletes in-season [75]. In their study, Vander Doelen and Jelly demonstrated that isometric exercises were found to reduce pain immediately, whereas eccentric exercises and PRP injections demonstrated good short-term as well as long-term results [76]. The authors concluded that the current evidence fails to suggest how to use conservative treatment options together to obtain the maximum benefit [76].

Regarding the difference between isometric and dynamic resistance exercises in reducing pain in patients with PT, a randomized crossover trial failed to identify the superiority of one method over the other [77]. They demonstrated a minor decrease in pain at the immediate post-exercise period, in both groups, with no essential difference in acute pain and pain sensitivity between the two groups.

Another issue that is worth mentioning is the role of progressive tendon-loading exercises (PTLE). Breda et al. in a randomized clinical trial including mostly patients suffering from chronic PT, showed better clinical outcomes with utilization of PLTE in comparison to eccentric exercises in a 2-year follow-up [78]. However, return to sports and patient satisfaction, although were better among patients of the PTLE group, did not achieve significant statistical difference.

#### Platelet-rich plasma (PRP) injections

PRP injections have been shown to be an effective treatment option for chronic PT [9, 27, 39–42, 54]. PRP injections with US guidance enable infiltration into the tendon with great accuracy [27]. This treatment stimulates soft tissue healing and improves tendon healing and remodeling [54]. Andriolo et al. suggest that multiple PRP injections could be the best option for patients with severe symptoms, or when other conservative treatments fail to alleviate chronic PT [9]. Patients with chronic PT and no previous treatment who received PRP injections had significantly better results in comparison to patients with chronic PT for the same duration, and previous failing therapies such as ethoxysclerol, corticosteroid and surgical treatment [41].

In a study comparing operative treatments versus PRP injections, it was found that even though operative treatments were shown to be safe and more effective, PRP infiltration is less invasive and could lead to tendon healing; therefore, it should be considered before an operative approach [41].

Kon E. et al. also indicated that multiple PRP injections in PT patients induced a statistically significant improvement at the end of the therapy and at the 6-month follow-up, with most of the patients being satisfied and able to return to their previous sport activity level [42].

Ferrero et al. assessed the effectiveness of US-guided autologous PRP injections in PT and Achilles tendinopathy with 20-day and 6-month follow-ups [27]. At the first follow-up, there was a non-significant improvement compared to baseline regarding VISA-P score, tendon thickness and the hypoechoic areas of the tendons. However, there was a significant improvement for all the above parameters at the second follow-up. Furthermore, in the same study, the intratendinous vascularity was significantly increased at both the first and the second follow-up. Thus, they concluded that US-guided PRP injections in PT patients lead to a significant improvement in both patellar tendon vascularity and symptoms [27].

According to Kaux et al. most of the existing studies assessed the effects of two or three successive PRP injections. In their study, the effectiveness of two PRP injections versus one PRP injection, in 20 PT patients who were divided into two groups, was compared [79]. They concluded that there was no significant difference between the two groups, and one PRP injection could be enough to treat PT in the short and medium terms [79].

In a study comparing PRP injections with focused ESWT for the treatment of PT, there was no significant difference between the study groups at the 2-month follow-up. However, PRP injections were shown to be more effective in the 6- and 12-months follow-ups [10].

Andriolo et al. indicated that even though eccentric exercises can be used as treatments in the short term, multiple PRP injections may offer more satisfactory results in the long term [9].

According to a recent study, PRP infiltrations with doses greater than 4 mL were found to have better results in the short term when combined with an exercise program for at least 6 weeks. However, in the long term, dry needling and skin-derived tenocyte-like cells were found to be more effective than PRP injections [80].

#### Cryotherapy

Cryotherapy is mostly used in PT patients for its short-term analgesic effect, but use is discouraged before participating in sports because there is a risk of re-injury [43, 44, 81]. Furthermore, cryotherapy reduces tissue metabolism, as well as swelling and pain in patients with acute inflammatory tendinopathies, by reducing the inflammatory response [81]. Although Mac Auley et al. indicated that, in the literature, there are considerable dissimilarities in the recommended duration of cryotherapy treatments [44], Bleakley et al. showed that applications of ice through a wet towel for 10-min periods are most effective [82].

#### Patellar strapping

Patellar strapping has historically been used widely, with controversial results; some studies indicate that it is an effective conservative treatment [45–51], and others have reported poor results [83–86]. Although Schwartz et al. demonstrated that most of the above studies evaluated patellofemoral pain in general [25], recent studies have been conducted to assess the effect of patellar strapping in athletes with PT [87, 88]. De Vries et al. conducted a randomized control trial to investigate the effect of patellar strapping and sports tape on pain in PT patients. They found that, interestingly, all the employed orthoses–patellar straps, sports tape and placebo taping–led to some pain-relief in the short term, as compared to controls [87]. Furthermore, in another study, De Vries et al. concluded that patellar strapping can reduce load on the patellar tendon [88].

#### NSAIDs

In the literature, the use of NSAIDs in chronic tendinopathy are controversial, because histologically, tendon tissue has few or no inflammatory cells [89]. Some writers have demonstrated that NSAIDs can help with tendon healing [52, 53], but others have indicated that NSAIDs may inhibit tendon cell migration and proliferation, impairing tendon healing [54, 90]. Furthermore, because NSAIDs reduce pain, patients sometimes ignore early symptoms, leading to inducing further damage to the tendon and consequently delaying healing. In addition, Tsai et al. claim that potential harms of NSAIDs—ulcers and renal impairment, for example—need to be taken into consideration [90].

#### Corticosteroids

Corticosteroids have been used as a treatment strategy for PT [25, 35, 54–58]. Corticosteroid injections were found to reduce pain, reduce swelling, and even improve the US findings of the tendon in severe tendonitis [25, 54]. However, the mechanisms behind their beneficial effects remain unclear [54, 58]. Some of the following studies concern the Achilles tendon; however, the results of corticosteroid injections in PT are similar [54]. Up to 82% of corticosteroid trials in tendons demonstrated adverse effects [91–93]. Some adverse effects are tendon rupture [57, 91, 92], atrophy [92] and decreased tendon strength [56, 93]. Although they are beneficial in the short term, they are not superior to other therapies in the long term [92].

#### Operative treatment

Many athletes with PT respond positively to non-operative treatment. However, some of them will need surgical treatment if conservative therapy fails [2, 25]. Although there is no gold standard management for PT, some authors have demonstrated that surgery is taken into consideration when athletes fail to improve after 6 months of conservative treatment [54, 94, 95]. Arthroscopic and open surgery have been reported as surgical treatment options [96, 97]. The success rates of arthroscopic procedures and open surgery were found to be 91% and 87%, respectively, according to a systematic review [97]. Time to return to pre-injury activity level was significantly less (3.9 months on average) after arthroscopic treatment in comparison to open surgery (8.3 months on average) [97]. In contrast, Aicale et al. reported that there as a similar rate of return to sports after arthroscopy and open treatment [54]. Thus, even though there are no clear guidelines in the literature about which technique is superior, arthroscopic techniques are more commonly preferred due to their less-invasive nature [97].

### Prevention

Prevention methods for PT are not well described in the literature. Nonetheless, balance and proprioception training and the use of a patellar strap are some methods used for the prevention of PT in sports.

Peters et al. claimed that long-term balance training can be used for the prevention of PT, but there was no positive outcome found when stretching exercises were used for this purpose [98]. This was also supported by Kraemer and Knobloch et al., who indicated that soccer-specific balance training was found to reduce PT with a dose–effect relationship between the duration of training and the incidence of PT [99]. Furthermore, in the study by Peters et al., the authors correlated prophylactic eccentric training protocols and stretching exercises with a high incidence of injury in asymptomatic athletes with PT abnormalities [98]. It is also not recommended to use in-season prophylactically eccentric exercise protocols in asymptomatic soccer players who exhibit pathologic imaging. This is supported also by Fredberg et al., who indicated that there was a higher risk of developing PT when this protocol was used [100]. In the same study, the prophylactic eccentric program was found to reduce the risk of developing US abnormalities in the patellar tendons of the athletes, but it was shown to have no positive effects on the risk of developing PT [100].

According to de Vries et al., small improvements in proprioception were found when athletes wore a patellar strap [88]. In addition, according to de Vries et al., patellar strapping improves proprioception of the symptomatic knee in athletes with poor proprioceptive acuity, mostly in patients with a relatively small knee girth, small tendon abnormalities and newly developed symptoms [86]. Thus, patellar strapping can play a role in injury prevention, because poor proprioception is believed to be a cause of injury and/or re-injury [88]. Kraemer and Knoblock et al. also support the hypothesis that proprioceptive training can also reduce the rehabilitation time from noncontact injuries such as PT [99].

## Conclusion

PT is a frequent clinical entity in athletes, especially in jumping sports such as volleyball and basketball. It is mostly diagnosed clinically, but US and MRI are also essential diagnostic tools. Risk factors for PT are the male gender, involvement in sports (particularly volleyball), playing at the national level, the position played in volleyball, training hours, playing surface, small patellar tendon lever and moment arm movements, hypoechoic areas and neovascularization displayed in US of the tendon, jump height, heavier load on the tendon and landing strategy of the athlete. Higher BMI as a risk factor has been supported by some authors but questioned by others. The screening of asymptomatic athletes, especially those with the aforementioned risk factors, using clinical examination, US, and VISA-P scores can be useful to predict which athletes will develop PT. Furthermore, patellar strapping can be used for prevention and rehabilitation purposes. Eccentric exercises are not advised as a prophylactic measurement during the training season for asymptomatic athletes with pathologic US. Balance and proprioception training are recommended in jumping sports because they are found to be good methods for prevention.

Even though the treatments for this condition can be non-operative and operative, non-operative treatment seems to be the first choice. Aicale et al. in their review agree that non-operative management is the mainstay of treatment, with about 10 percent of refractory cases ending up in surgical treatment [101]. Conservative treatment consists of eccentric exercises, PRP injections–with PLTE showing also promising outcomes. The results of combinations of these two methods with other non-operative modalities are not well documented in the literature. Noteworthy, a recent systematic review found that conservative treatment with load management and physical exercise can result in favorable clinical outcomes in the short and long-term periods [102]. Even though there is no gold standard protocol described in the literature, when athletes treated with conservative treatment fail to improve after 6 months of therapy, surgery is the next recommended step, with arthroscopic techniques being most preferable because of their minimally invasive nature, possibly enabling a faster return to sport.

This review has some limitations that should be mentioned. First of all, studies with small samples were included, and this may be considered less reliable and representative of the athletes’ population. Additionally, even though some studies had a great number of participants, they were conducted using online questionaries and that may question the reliability of their results. Furthermore, the results in many studies come in contrast with other studies, especially in the field of conservative treatment. However, it represents a thorough overview of the available literature that could be of remarkable assistance to clinicians who deal with this entity. Undeniably, more studies and clinical trials are necessary to draw reliable conclusions.

## Data Availability

Full text articles that were extracted during research are stored in the department’s flash drive.

## References

[1] Peers KHE, Lysens RJJ (2005). Patellar tendinopathy in athletes: current diagnostic and therapeutic recommendations. Sports Med.

[2] Muaidi QI (2020). Rehabilitation of patellar tendinopathy. J Musculoskelet Neuronal Interact.

[3] Kettunen JA, Kvist M, Alanen E, Kujala UM (2002). Long-term prognosis for jumper’s knee in male athletes. A prospective follow-up study. Am J Sports Med.

[4] Lian OB, Engebretsen L, Bahr R (2005). Prevalence of jumper’s knee among elite athletes from different sports: a cross-sectional study. Am J Sports Med.

[5] Zwerver J, Bredeweg SW, van den Akker-Scheek I (2011). Prevalence of Jumper’s knee among nonelite athletes from different sports: a cross-sectional survey. Am J Sports Med.

[6] De Vries AJ, Koolhaas W, Zwerver J, Diercks RL, Nieuwenhuis K, Van Der Worp H (2017). The impact of patellar tendinopathy on sports and work performance in active athletes. Res Sports Med.

[7] Cook JL, Rio E, Purdam CR, Docking SI (2016). Revisiting the continuum model of tendon pathology: what is its merit in clinical practice and research?. Br J Sports Med.

[8] de Vries AJ, van der Worp H, Diercks RL, van den Akker-Scheek I, Zwerver J (2015). Risk factors for patellar tendinopathy in volleyball and basketball players: a survey-based prospective cohort study. Scand J Med Sci Sports.

[9] Andriolo L, Altamura SA, Reale D, Candrian C, Zaffagnini S, Filardo G (2019). Nonsurgical treatments of patellar tendinopathy: multiple injections of platelet-rich plasma are a suitable option: a systematic review and meta-analysis. Am J Sports Med.

[10] Vetrano M, Castorina A, Vulpiani MC, Baldini R, Pavan A, Ferretti A (2013). Platelet-rich plasma versus focused shock waves in the treatment of jumper’s knee in athletes. Am J Sports Med.

[11] van der Worp H, van Ark M, Zwerver J, van den Akker-Scheek I (2012). Risk factors for patellar tendinopathy in basketball and volleyball players: a cross-sectional study. Scand J Med Sci Sports.

[12] Lian Ø, Refsnes P-E, Engebretsen L, Bahr R (2003). Performance characteristics of volleyball players with patellar tendinopathy. Am J Sports Med.

[13] Sprague AL, Smith AH, Knox P, Pohlig RT, Grävare SK (2018). Modifiable risk factors for patellar tendinopathy in athletes: a systematic review and meta-analysis. Br J Sports Med.

[14] Crossley KM, Thancanamootoo K, Metcalf BR, Cook JL, Purdam CR, Warden SJ (2007). Clinical features of patellar tendinopathy and their implications for rehabilitation. J Orthop Res.

[15] Gaida JE, Cook JL, Bass SL, Austen S, Kiss ZS (2004). Are unilateral and bilateral patellar tendinopathy distinguished by differences in anthropometry, body composition, or muscle strength in elite female basketball players?. Br J Sports Med.

[16] Witvrouw E, Bellemans J, Lysens R, Danneels L, Cambier D (2001). Intrinsic risk factors for the development of patellar tendinitis in an athletic population. A two-year prospective study. Am J Sports Med.

[17] Lian O, Engebretsen L, Ovrebø RV, Bahr R (1996). Characteristics of the leg extensors in male volleyball players with jumper’s knee. Am J Sports Med.

[18] van der Worp H, Zwerver J, Kuijer PPFM, Frings-Dresen MHW, van den Akker-Scheek I (2011). The impact of physically demanding work of basketball and volleyball players on the risk for patellar tendinopathy and on work limitations. J Back Musculoskelet Rehabil.

[19] Cook JL, Bass SL, Black JE (2007). Hormone therapy is associated with smaller Achilles tendon diameter in active post-menopausal women. Scand J Med Sci Sports.

[20] Bahr R, Reeser JC (2003). Injuries among world-class professional beach volleyball players. The Fédération Internationale de Volleyball beach volleyball injury study. Am J Sports Med.

[21] Dan MJ, McMahon J, Parr WCH, Broe D, Lucas P, Cross M (2018). Evaluation of intrinsic biomechanical risk factors in patellar tendinopathy: a retrospective radiographic case-control series. Orthop J Sports Med.

[22] Malliaras P, Cook JL, Kent PM (2007). Anthropometric risk factors for patellar tendon injury among volleyball players. Br J Sports Med.

[23] Bisseling RW, Hof AL, Bredeweg SW, Zwerver J, Mulder T (2007). Relationship between landing strategy and patellar tendinopathy in volleyball. Br J Sports Med.

[24] Visnes H, Tegnander A, Bahr R (2015). Ultrasound characteristics of the patellar and quadriceps tendons among young elite athletes. Scand J Med Sci Sports.

[25] Schwartz A, Watson JN, Hutchinson MR (2015). Patellar tendinopathy. Sports Health.

[26] Cook JL, Khan KM, Maffulli N, Purdam C (2000). Overuse tendinosis, not tendinitis part 2: applying the new approach to patellar tendinopathy. Phys Sportsmed.

[27] Ferrero G, Fabbro E, Orlandi D, Martini C, Lacelli F, Serafini G (2012). Ultrasound-guided injection of platelet-rich plasma in chronic Achilles and patellar tendinopathy. J Ultrasound.

[28] Khan KM, Bonar F, Desmond PM, Cook JL, Young DA, Visentini PJ (1996). Patellar tendinosis (jumper’s knee): findings at histopathologic examination, US, and MR imaging. Victorian institute of sport tendon study group. Radiology.

[29] Campbell RS, Grainger AJ (2001). Current concepts in imaging of tendinopathy. Clin Radiol.

[30] Figueroa D, Figueroa F, Calvo R (2016). Patellar tendinopathy: diagnosis and treatment. J Am Acad Orthop Surg.

[31] Terslev L, Qvistgaard E, Torp-Pedersen S, Laetgaard J, Danneskiold-Samsøe B, Bliddal H (2001). Ultrasound and Power Doppler findings in jumper’s knee — preliminary observations. Eur J Ultrasound.

[32] Weinberg EP, Adams MJ, Hollenberg GM (1998). Color doppler sonography of patellar tendinosis. AJR Am J Roentgenol.

[33] Weatherall PT, Crues JV (1995). Musculotendinous injury. Magn andom Imaging Clin N Am.

[34] Warden SJ, Brukner P (2003). Patellar tendinopathy. Clin Sports Med.

[35] Fredberg U, Bolvig L (2002). Significance of ultrasonographically detected asymptomatic tendinosis in the patellar and achilles tendons of elite soccer players: a longitudinal study. Am J Sports Med.

[36] Fazekas ML, Sugimoto D, Cianci A, Minor JL, Corrado GD, d’Hemecourt PA (2018). Ultrasound examination and patellar tendinopathy scores in asymptomatic college jumpers. Phys Sportsmed.

[37] Gisslén K, Gyulai C, Nordström P, Alfredson H (2007). Normal clinical and ultrasound findings indicate a low risk to sustain jumper’s knee patellar tendinopathy: a longitudinal study on Swedish elite junior volleyball players. Br J Sports Med.

[38] Visnes H, Bahr R (2007). The evolution of eccentric training as treatment for patellar tendinopathy (jumper’s knee): a critical review of exercise programmes. Br J Sports Med.

[39] Bowman KFJ, Muller B, Middleton K, Fink C, Harner CD, Fu FH (2013). Progression of patellar tendinitis following treatment with platelet-rich plasma: case reports. Knee Surg Sports Traumatol Arthrosc.

[40] Ferretti A, Ippolito E, Mariani P, Puddu G (1983). Jumper’s knee. Am J Sports Med.

[41] Gosens T, Den Oudsten BL, Fievez E, van’t Spijker P, Fievez A (2012). Pain and activity levels before and after platelet-rich plasma injection treatment of patellar tendinopathy: a prospective cohort study and the influence of previous treatments. Int Orthop.

[42] Kon E, Filardo G, Delcogliano M, Presti ML, Russo A, Bondi A (2009). Platelet-rich plasma: new clinical application: a pilot study for treatment of jumper’s knee. Injury.

[43] Rivenburgh DW (1992). Physical modalities in the treatment of tendon injuries. Clin Sports Med.

[44] MacAuley D (2001). Do textbooks agree on their advice on ice?. Clin J Sport Med.

[45] Adirim TA, Cheng TL (2003). Overview of injuries in the young athlete. Sports Med.

[46] Bohnsack M, Halcour A, Klages P, Wilharm A, Ostermeier S, Rühmann O (2008). The influence of patellar bracing on patellar and knee load-distribution and kinematics: an experimental cadaver study. Knee Surg Sports Traumatol Arthrosc.

[47] Braverman SE (2002). Orthotics for the fighting force. Phys Med Rehabil Clin N Am.

[48] Gerrard DF (1998). External knee support in rugby union. Effectiveness of bracing and taping. Sports Med.

[49] Greene BL (2002). Physical therapist management of fluoroquinolone-induced Achilles tendinopathy. Phys Ther.

[50] Levine J, Splain S (1979). Use of the infrapatella strap in the treatment of patellofemoral pain. Clin Orthop Relat Res.

[51] D’hondt NE, Struijs PA, Kerkhoffs GM, Verheul C, Lysens R, Aufdemkampe G (2002). Orthotic devices for treating patellofemoral pain syndrome. Cochrane Database Syst Rev.

[52] Forslund C, Bylander B, Aspenberg P (2003). Indomethacin and celecoxib improve tendon healing in rats. Acta Orthop Scand.

[53] Weiler JM (1992). Medical modifiers of sports injury. The use of nonsteroidal anti-inflammatory drugs (NSAIDs) in sports soft-tissue injury. Clin Sports Med..

[54] Aicale R, Bisaccia RD, Oliviero A, Oliva F, Maffulli N (2020). Current pharmacological approaches to the treatment of tendinopathy. Expert Opin Pharmacother.

[55] Fredberg U, Bolvig L, Pfeiffer-Jensen M, Clemmensen D, Jakobsen BW, Stengaard-Pedersen K (2004). Ultrasonography as a tool for diagnosis, guidance of local steroid injection and together with pressure algometry, monitoring of the treatment of athletes with chronic jumper’s knee and Achilles tendinitis: a randomized, double-blind, placebo-controlled study. Scand J Rheumatol.

[56] Kennedy JC, Willis RB (1976). The effects of local steroid injections on tendons: a biomechanical and microscopic correlative study. Am J Sports Med.

[57] Kongsgaard M, Aagaard P, Kjaer M, Magnusson SP (2005). Structural Achilles tendon properties in athletes subjected to different exercise modes and in Achilles tendon rupture patients. J Appl Physiol.

[58] Paavola M, Kannus P, Järvinen TAH, Järvinen TLN, Józsa L, Järvinen M (2002). Treatment of tendon disorders. Is there a role for corticosteroid injection?. Foot Ankle Clin.

[59] Magra M, Maffulli N (2008). Genetic aspects of tendinopathy. J Sci Med Sport.

[60] Orchard J, Massey A, Brown R, Cardon-Dunbar A, Hofmann J (2008). Successful management of tendinopathy with injections of the MMP-inhibitor aprotinin. Clin Orthop Relat Res.

[61] Alfredson H, Ohberg L (2005). Neovascularisation in chronic painful patellar tendinosis—promising results after sclerosing neovessels outside the tendon challenge the need for surgery. Knee Surg Sports Traumatol Arthrosc.

[62] Alfredson H, Ohberg L (2005). Sclerosing injections to areas of neo-vascularisation reduce pain in chronic Achilles tendinopathy: a double-blind randomized controlled trial. Knee Surg Sports Traumatol Arthrosc.

[63] Hoksrud A, Bahr R (2011). Ultrasound-guided sclerosing treatment in patients with patellar tendinopathy (jumper’s knee). 44-month follow-up. Am J Sports Med.

[64] Hoksrud A, Ohberg L, Alfredson H, Bahr R (2006). Ultrasound-guided sclerosis of neovessels in painful chronic patellar tendinopathy: a randomized controlled trial. Am J Sports Med.

[65] Scott A, Lian Ø, Bahr R, Hart DA, Duronio V (2008). VEGF expression in patellar tendinopathy: a preliminary study. Clin Orthop Relat Res.

[66] Willberg L, Sunding K, Forssblad M, Fahlström M, Alfredson H (2011). Sclerosing polidocanol injections or arthroscopic shaving to treat patellar tendinopathy/jumper’s knee? A randomized controlled study. Br J Sports Med.

[67] Paoloni JA, Appleyard RC, Nelson J, Murrell GAC (2005). Topical glyceryl trinitrate application in the treatment of chronic supraspinatus tendinopathy: a randomized, double-blinded, placebo-controlled clinical trial. Am J Sports Med.

[68] Chung B, Wiley JP (2002). Extracorporeal shockwave therapy: a review. Sports Med.

[69] Young MA, Cook JL, Purdam CR, Kiss ZS, Alfredson H (2005). Eccentric decline squat protocol offers superior results at 12 months compared with traditional eccentric protocol for patellar tendinopathy in volleyball players. Br J Sports Med.

[70] Jonsson P, Alfredson H (2005). Superior results with eccentric compared to concentric quadriceps training in patients with jumper’s knee: a prospective randomized study. Br J Sports Med.

[71] Visnes H, Hoksrud A, Cook J, Bahr R (2005). No effect of eccentric training on jumper’s knee in volleyball players during the competitive season: a randomized clinical trial. Clin J Sport Med.

[72] Saithna A, Gogna R, Baraza N, Modi C, Spencer S (2012). Eccentric exercise protocols for patella tendinopathy: should we really be withdrawing athletes from sport? A systematic review. Open Orthop J.

[73] Cannell LJ, Taunton JE, Clement DB, Smith C, Khan KM (2001). A randomized clinical trial of the efficacy of drop squats or leg extension/leg curl exercises to treat clinically diagnosed jumper’s knee in athletes: pilot study. Br J Sports Med.

[74] Bahr R, Fossan B, Løken S, Engebretsen L (2006). Surgical treatment compared with eccentric training for patellar tendinopathy (Jumper’s Knee). A randomized, controlled trial. J Bone Joint Surg Am.

[75] van Ark M, Cook JL, Docking SI, Zwerver J, Gaida JE, van den Akker-Scheek I (2016). Do isometric and isotonic exercise programs reduce pain in athletes with patellar tendinopathy in-season? A andomized clinical trial. J Sci Med Sport.

[76] Vander Doelen T, Jelley W (2020). Non-surgical treatment of patellar tendinopathy: a systematic review of randomized controlled trials. J Sci Med Sport.

[77] Holden S, Lyng K, Graven-Nielsen T, Riel H, Olesen JL, Larsen LH (2020). Isometric exercise and pain in patellar tendinopathy: a randomized crossover trial. J Sci Med Sport.

[78] Breda SJ, Oei EHG, Zwerver J, Visser E, Waarsing E, Krestin GP (2021). Effectiveness of progressive tendon-loading exercise therapy in patients with patellar tendinopathy: a randomized clinical trial. Br J Sports Med.

[79] Kaux JF, Croisier JL, Forthomme B, Le Goff C, Buhler F, Savanier B (2016). Using platelet-rich plasma to treat jumper’s knees: exploring the effect of a second closely-timed infiltration. J Sci Med Sport.

[80] López-Royo MP, Ortiz-Lucas M, Gómez-Trullén EM, Herrero P (2020). The effectiveness of minimally invasive techniques in the treatment of patellar tendinopathy: a systematic review and meta-analysis of randomized controlled trials. Evid Based Complement Alternat Med.

[81] Wilson JJ, Best TM (2005). Common overuse tendon problems: A review and recommendations for treatment. Am Fam Physician.

[82] Bleakley C, McDonough S, MacAuley D (2004). The use of ice in the treatment of acute soft-tissue injury: a systematic review of randomized controlled trials. Am J Sports Med.

[83] Finestone A, Radin EL, Lev B, Shlamkovitch N, Wiener M, Milgrom C (1993). Treatment of overuse patellofemoral pain. Prospective randomized controlled clinical trial in a military setting. Clin Orthop Relat Res.

[84] Miller MD, Hinkin DT, Wisnowski JW (1997). The efficacy of orthotics for anterior knee pain in military trainees. A preliminary report. Am J Knee Surg.

[85] Villar RN (1985). Patellofemoral pain and the infrapatellar brace. A military view. Am J Sports Med.

[86] de Vries AJ, van den Akker-Scheek I, Haak SL, Diercks RL, van der Worp H, Zwerver J (2017). Effect of a patellar strap on the joint position sense of the symptomatic knee in athletes with patellar tendinopathy. J Sci Med Sport.

[87] de Vries A, Zwerver J, Diercks R, Tak I, van Berkel S, van Cingel R (2016). Effect of patellar strap and sports tape on pain in patellar tendinopathy: a randomized controlled trial. Scand J Med Sci Sports.

[88] de Vries AJ, van den Akker-Scheek I, Diercks RL, Zwerver J, van der Worp H (2016). The effect of a patellar strap on knee joint proprioception in healthy participants and athletes with patellar tendinopathy. J Sci Med Sport.

[89] Ribbans WJ, Collins M (2013). Pathology of the tendo Achillis: do our genes contribute?. Bone Joint J.

[90] Tsai W-C, Hsu C-C, Chou S-W, Chung C-Y, Chen J, Pang J-HS (2007). Effects of celecoxib on migration, proliferation and collagen expression of tendon cells. Connect Tissue Res.

[91] Chen S-K, Lu C-C, Chou P-H, Guo L-Y, Wu W-L (2009). Patellar tendon ruptures in weight lifters after local steroid injections. Arch Orthop Trauma Surg.

[92] Hart L (2011). Corticosteroid and other injections in the management of tendinopathies: a review. Clin J Sport Med.

[93] Haraldsson BT, Langberg H, Aagaard P, Zuurmond A-M, van El B, Degroot J (2006). Corticosteroids reduce the tensile strength of isolated collagen fascicles. Am J Sports Med.

[94] Everhart JS, Cole D, Sojka JH, Higgins JD, Magnussen RA, Schmitt LC (2017). Treatment options for patellar tendinopathy: a systematic review. Arthroscopy.

[95] Schmitz C, Császár NBM, Milz S, Schieker M, Maffulli N, Rompe J-D (2015). Efficacy and safety of extracorporeal shock wave therapy for orthopedic conditions: a systematic review on studies listed in the PEDro database. Br Med Bull.

[96] Dan M, Phillips A, Johnston RV, Harris IA (2019). Surgery for patellar tendinopathy (jumper’s knee). Cochrane Database Syst Rev.

[97] Brockmeyer M, Diehl N, Schmitt C, Kohn DM, Lorbach O (2015). Results of surgical treatment of chronic patellar tendinosis (jumper’s knee): a systematic review of the literature. Arthroscopy.

[98] Peters JA, Zwerver J, Diercks RL, Elferink-Gemser MT, van den Akker-Scheek I (2016). Preventive interventions for tendinopathy: a systematic review. J Sci Med Sport.

[99] Kraemer R, Knobloch K (2009). A soccer-specific balance training program for hamstring muscle and patellar and achilles tendon injuries: an intervention study in premier league female soccer. Am J Sports Med.

[100] Fredberg U, Bolvig L, Andersen NT (2008). Prophylactic training in asymptomatic soccer players with ultrasonographic abnormalities in Achilles and patellar tendons: the Danish Super League Study. Am J Sports Med.

[101] Aicale R, Oliviero A, Maffulli N (2020). Management of Achilles and patellar tendinopathy: what we know, what we can do. J Foot Ankle Res.

[102] Núñez-Martínez P, Hernández-Guillen D (2022). Management of patellar tendinopathy through monitoring, load control, and therapeutic exercise: a systematic review. J Sport Rehabil.

